# Survival of irradiated recipient mice after transplantation of bone marrow from young, old and “early aging” mice

**DOI:** 10.18632/aging.100867

**Published:** 2015-12-30

**Authors:** Ian Guest, Zoran Ilic, Heidi Scrable, Stewart Sell

**Affiliations:** ^1^ Wadsworth Center, New York State Department of Health, Albany, NY 12201, USA; ^2^ Laboratory Medicine and Pathology, Mayo Clinic, Rochester, MN 55905, USA

**Keywords:** p53, p44, bone marrow transplantation, aging

## Abstract

Bone marrow transplantation is used to examine survival, hematopoietic stem cell function and pathology in recipients of young and old wild type bone marrow derived stem cells (BMDSCs) as well as cells from p53-based models of premature aging. There is no difference in the long term survival of recipients of 8 week-old p53+/m donor cells compared to recipients of 8 week-old wild-type (WT) donor cells (70 weeks) or of recipients of 16–18 weeks-old donor cells from either p53+/m or WT mice. There is shorter survival in recipients of older versus younger WT donor bone marrow, but the difference is only significant when comparing 8 and 18 week-old donors. In the p44-based model, short term survival/engraftment is significantly reduced in recipients of 11 month-old p44 donor cells compared to 4 week-old p44 or wild type donor cells of either age; mid-life survival at 40 weeks is also significantly less in recipients of p44 cells. BMDSCs are readily detectable within recipient bone marrow, lymph node, intestinal villi and liver sinusoids, but not in epithelial derived cells. These results indicate that recipients of young BMDSCs may survive longer than recipients of old bone marrow, but the difference is marginal at best.

## INTRODUCTION

Bone marrow derived stem cells (BMDSCs) were initially studied for their role in hematopoiesis but in the last 15–20 years the role of these stem cells in regeneration and repair of other tissues and in aging has been an active area of research. In the 1950′s it was found that lethally irradiated recipients could be rescued by transplantation of spleen or bone marrow (BM) cells [1–3]. In the 1970′s, Harrison et al. demonstrated through serial BM transplantation experiments that murine BMDSCs could survive and produce progeny in the recipients for more than 8 years [4, 5]. The widely expressed protein p53 is a critical participant in tumor suppression and more than 50% of human cancers contain mutations in p53. Though the functions of this protein in cancer have been extensively studied, recent work suggests a role for p53 in prolonging life [6–8]. To test the hypothesis that BMDSCs contribute to the stem cell population of other organs and in this way restore tissue stem cells and delay organismal aging, we studied the effect of bone marrow transplantation using young and old donors and p53-based early aging mouse models. We theorized that if BMDSCs play a role in the aging process, then transplantation of BM from early aging donors into lethally irradiated wild-type recipients should induce premature aging, and conversely, transplantation of bone marrow (BM) from young wild-type donors should extend the lifespan of recipients with early aging phenotypes. Unexpectedly, in these studies, control groups of C57BL/6 mice receiving young wild-type bone marrow did not survive significantly longer than mice receiving old wild type bone marrow. Due to difficulties with the p53+/m model [6], we switched to the p44 model of Maier et al. [9]. In this study we found that the ICR strain of mice receiving either young or old p44 bone marrow had a significant decrease in both short term survival and in mid-life survival as compared to ICR mice receiving comparable young and old wild-type BM, suggesting a deficit in short term hematopoietic stem cells (ST-HSCs) in p44 mice.

## RESULTS

### Long-term survival studies; p53+/m aging model

**Figure 1:**
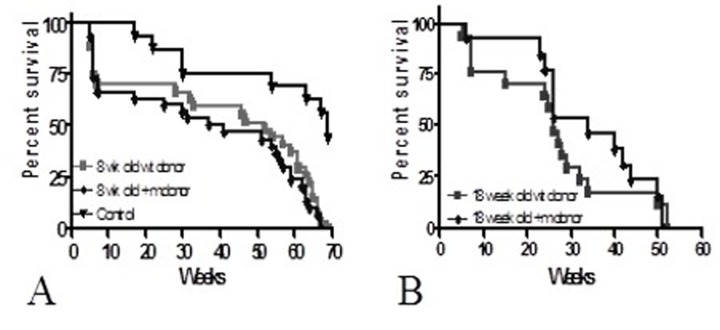
Long-term survival of C57Bl/6 recipients (**A**) Four-week old female C57Bl/6 mice were lethally irradiated and transplanted with 2 × 10^6^ BMCs from 8-week old +/m or wild-type donors (n=20/group). (**B**) Four-week old female C57Bl/6 mice were lethally irradiated and transplanted with 2 × 10^6^ BMCs from 18-week old +/m (n=13) or wild-type donors (n=17). No differences were detected between the two groups of recipients suggesting that lifespan was not reduced by transplantation of +/m BMCs.

To test the hypothesis that premature aging could be induced in wild-type recipients of +/m bone marrow, we transplanted four-week old lethally irradiated wild-type female C57Bl/6 mice with 2 × 10^6^ BM from 8 week old p53+/m male C57Bl/6 mice and a control group with 8 week old wild-type male bone marrow (n=20/group). An unirradiated, untransplanted control group was also included (n=12). Long-term survival was not significantly different between both transplanted groups (lower curves Figure 1A); mean time to death was 46.6+/−12.9 weeks in the group that received wild-type BM vs. 43.6+/−14.7 weeks in the group that received p53+/m BM (p=NS). For both groups, maximum survival was approximately 70 weeks (p=NS). Although overall growth was also similar, recipients of +/m BM achieved maximal weight approximately five weeks sooner that wild type (43.8 weeks +/m vs. 47.2 weeks wild type, p<0.0001), suggesting that the recipients of the +/m BM may be reaching “middle age” before the recipients of wild-type BM. In a second experiment, we investigated the effect of transplanting mature BMDSCs on long-term survival. Four-week old lethally irradiated female C57Bl/6 recipients received BM from 18-week old wild-type C57Bl/6 (n=17 recipients) or +/m (n=13 recipients) male donors. No statistically significant differences in long-term survival were detected between the recipients of wild-type or +m BM (Figure 1B).

### Young vs. old bone marrow

**Figure 2:**
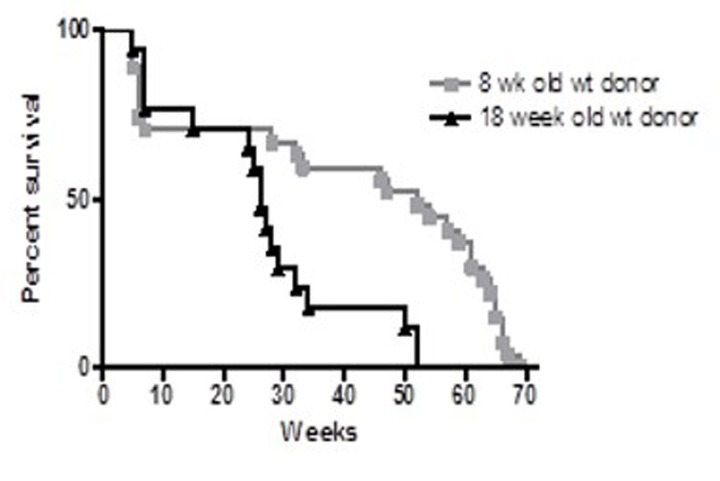
Survival proportions of irradiated C57Bl/6 female recipients Four week-old female recipients were lethally irradiated with (1050 rads) and transplanted with 2 × 10^6^ BM from 8 week-old (squares, n = 20) or 18 week-old (triangles, n = 17) male C57Bl/6 donors. Recipients of older (18 week-old) BM exhibited reduced longevity compared to recipients of younger (8 week) BM (p<0.001).

When the control groups from these two experiments were compared, the long-term survival was significantly longer in recipients of the younger (8-week old) BM than in recipients of BM from older (four-month old) donors (p<0.001) (Figure 2). These results suggest BMDSCs from older donors may be defective in their long-term repopulation capacity. To confirm this observation, an additional experiment was performed comparing survival of recipients of young (7 weeks) and old (27 months) bone marrow.

### Transplantation of young and old male bone marrow to female recipients

**Figure 3:**
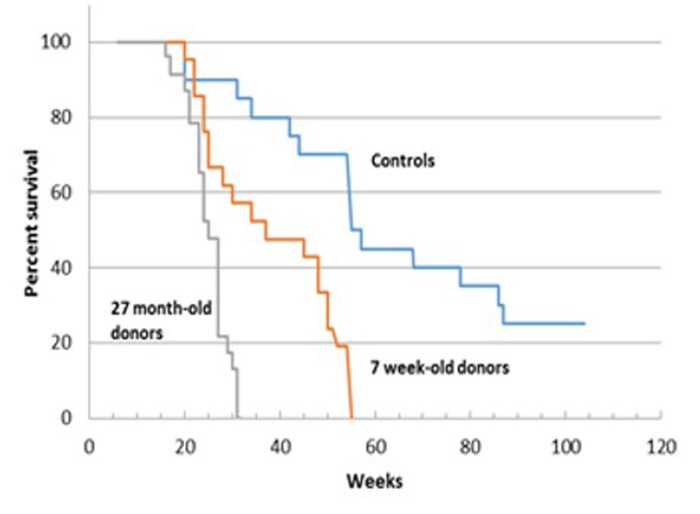
Survival analysis of C57BL/6 female mice receiving BM from young (7 week-old) and old (27 month-old) male donors

Survival of female C57Bl/6 mice transplanted with bone marrow cells (BMCs) from young (7 weeks) and old (27 months) male C57Bl/6 donors is shown in Figure 3. Originally 27 mice were included in each group. Twenty three mice that received BM from 27 week old donors and 21 recipient mice that received BM from 7 week old donors survived the 4 weeks post transplantation. Untransplanted control survival monitoring began at 6 weeks of age and the experiment was terminated at 104 weeks. Both recipient groups had significantly shorter survival than control un-irradiated groups. From first review of the Kaplan-Meier survival curves, the survival of recipients of 27 month-old BM appeared shorter than that of recipients of 7 week old BM (e.g. maximum lifespan was 28 weeks and 55 weeks, respectively). However, statistical analysis failed to confirm a significant difference between these groups. (See Supplement 1 for statistical analysis.).

### Pathology

A summary of pathologic findings in control and recipient mice is given in Table 2, photomicrographs of lesions in Supplement 2 and a listing of autopsy findings in Supplemental Tables 1 (control); 2 (old recipients) and 3 (young recipients) as well as histopathologic grading of lesions. Chronic hepatitis was found in all recipients of old BM. Macrophage pneumonia in recipients of both young and old marrow, and glomerulosclerosis in all recipients of young BM after 10 months of age. In general the listed lesions increased dramatically with age. For example, older recipients of 27 month old BM had severe hepatitis and macrophage pneumonia, whereas older recipients of young BM had severe glomerulosclerosis [9a]. Barbering (excessive grooming) is frequent in female C57Bl.6 mice and begins during puberty [10]; although not necessarily fatal, animal welfare protocols may require euthanasia when barbering results in chronic skin lesions. Macrophage pneumonia occurs in 13% of normally aging mice and “nephropathy” in 100% [11]. The reason for the 100% incidence of chronic hepatitis in recipients of old BM is not clear, but the severity of hepatitis increases with age, so that older mice in this group have extensive destruction of the liver (Suppl. Figure 1). Similarly, glomerulosclerosis occurs in 100% of older recipients of young BM and leads to hyalinization of most of the glomeruli. Macrophage pneumonia also increases in severity with age in both transplant groups, but not in control groups even after 27 weeks of age. Few lesions are seen in the control mice, which were euthanized to provide age matched controls for the transplanted mice [12].

**Table 1:** Survival in months of recipients of young or old bone marrow

	Controls*	Old BM Recipients	Young BM recipients
Number	12	12	20
Mean	16.5	7.9	11.0
Range	12–27	7.5–9	7–14.5

* Mice in control group were euthanized to obtain age matched tissues.

**Table 2:** Frequency of pathologic lesions in controls and mice receiving 7 week-old or 27month-old BM

	Total No.	Barbering*	Hepatitis	Macrophage pneumonia	Glomerulo-sclerosis	Focal lymphoid aggregates
Controls	20	4	0	1	0	4
Old BM	23	0	23	23	0	2
Young BM	21	3	0	21	14	4

* Frequency higher in control mice leading to early euthanasia of 2 mice.

### Staining for donor derived cells

To determine the cellular origin of various tissue cells after BM transplantation of 7 week-old or 27 month-old donors, we labeled for GFP+ and Y chromosome in the tissue of female mice transplanted with male GFP+ BM. As shown in Supplement 3 there is strong labeling for both markers in the bone marrow, the medulla of the lymph nodes and in the stromal cells of the intestinal villi and the sinusoidal cells (Kupffer cells) of the liver. However, there is no convincing labeling of any epithelial cells.

### Change of p53 early aging model

**Figure 4:**
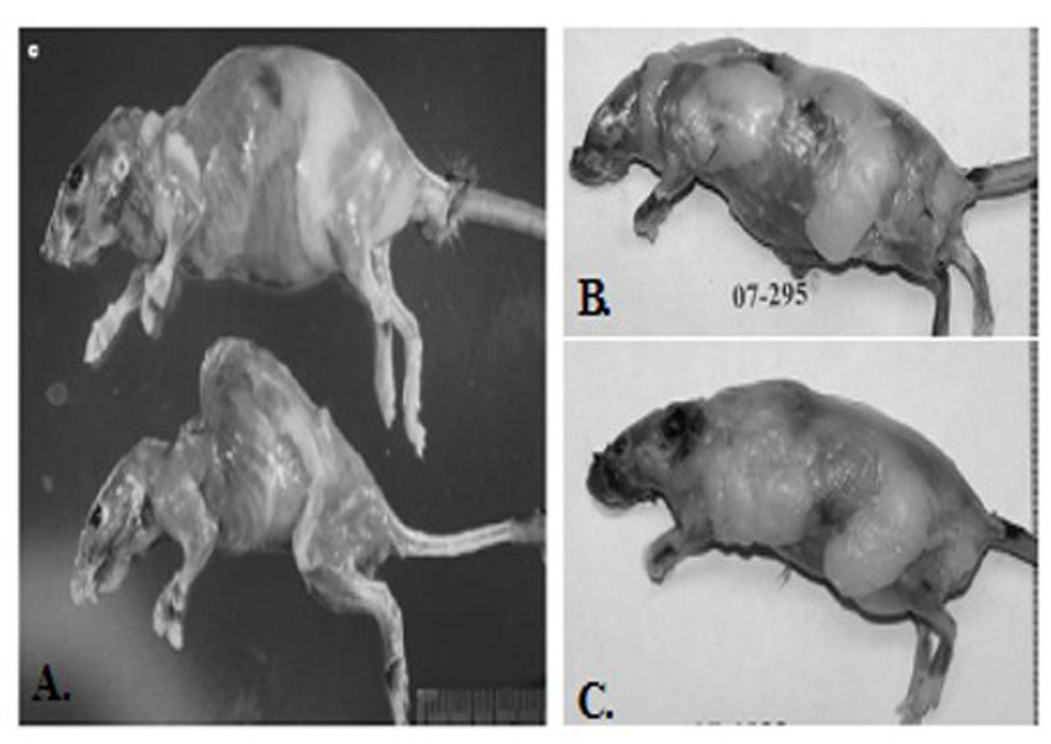
Comparison of phenotypes of p53+/m as originally reported in Tyner et al (2002) and those maintained by the Sell group (**A**) Top: Representative 20-month-old skinned *p53*^+/+^ female mouse (top) and age- and sex-matched *p53*^+/m^ mouse (bottom). Pronounced lordokyphosis, loss of body mass, muscle atrophy and loss of adipose tissue is evident in the *p53*^+/m^ mouse (**B** and** C**). Representative of a skinned 20 month-old (**B**) and 8 month-old male (**C**) mouse. Both mice possess excessive amounts of adipose tissue and do not exhibit lordokyphosis.

**Figure 5:**
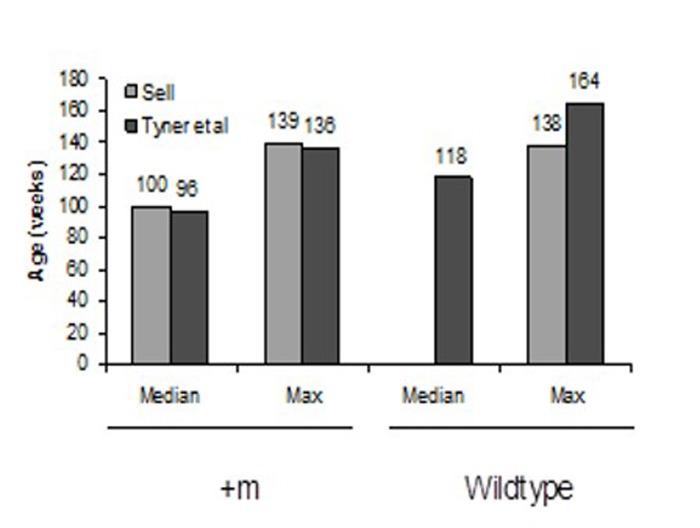
Mean and maximum lifespans of “Sell” and “Tyner et al” mice p53+/m and wildtype mice Mean and maximum lifespans of Sell and Tyner mice are nearly identical.

We planned to use the p53+/m mice as recipients of BM from wild-type donors in order to test the hypothesis that this would extend the lifespan of the +/m mice. However, we could not obtain enough age-matched +/m mice to serve as recipients. Although the +/m allele should segregate in a Mendelian fashion, only one out of every 8 to 12 mice were positive for +/m allele. Furthermore, while Tyner et al. reported a maximum mean weight of approximately 25 grams, the weight of the +/m mice that were bred in our facility reached a mean maximum weight of 50 grams by four months of age. Additionally, the mice bred in our facility did not exhibit the reductions in adipose tissue deposition, muscle mass or lordokyphosis as reported in Tyner et al. [6] (Figure 4A-B). However, the reported mean and maximum life-spans reported in Tyner et al. and those bred by us were almost identical (Figure 5), demonstrating that the early age of death was maintained in our colony, but the aging phenotype was not.

The differences in the weights of our mice and those reported in Tyner et al. are striking, but difficult to explain. In the work published by Tyner et al. [6] the +/m mice had only been backcrossed to C57Bl/6 for 2 generations and were on a C57BL/6–129/Sv background, so it is possible that as the mice were further backcrossed onto a C57Bl/6 background, strain differences emerged. Another possible explanation may lie in the recent discovery that C57Bl/6 mice from Jackson Laboratories carry a deletion of exons 7 to 11 in the gene encoding for nicotinamide nucleotide transhydrogenase (Nnt), which catalyzes the interconversion of NADH and NADPH in the mitochondria [13, 14]. Freeman et al. [14, 15] demonstrated that the Nnt deficiency results in defective insulin secretion and inappropriate glucose homeostasis in male C57BL/6J mice. It is possible that the combination of defective IGF signaling due to the presence of the +/m allele with the Nnt deletion is responsible for the observation that our +/m are twice the mass as those reported by Tyner et al. [6]. Finally, because our mice were re-derived at Taconic Farms prior to delivery to our animal facility, it is possible that there are differences in the C57Bl/6J backgrounds used by Dr. Donehower and those at Taconic Farms.

Due to the problems with the breeding and the discrepancies between the reported +/m phenotypes and those observed in our facility, we elected to change the premature aging model from +/m to the p44 early aging mouse model.

### p44 premature aging model: short-term studies

**Figure 6:**
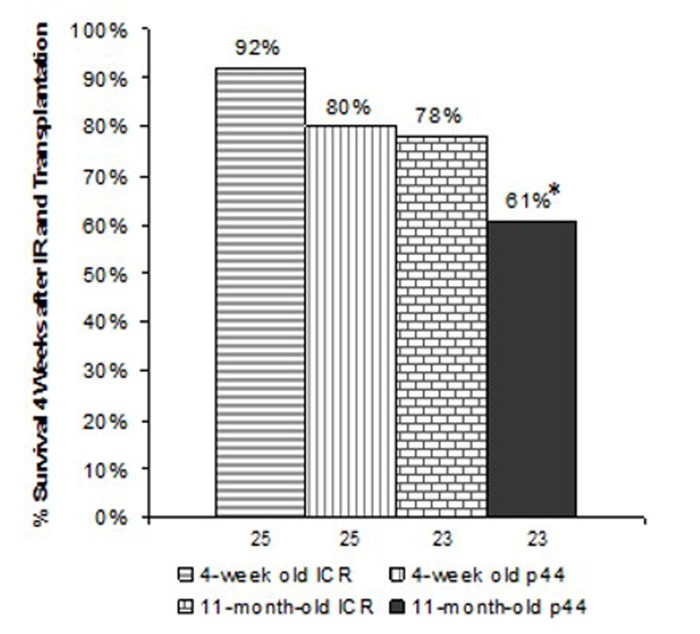
Survival 4-weeks post-irradiation and transplantation Numbers indicate the number of animals initially irradiated and transplanted. *indicates p<0.01 compared to recipients who had received BMCs from 4-week old ICR male donors as determined by chi-square test.

**Figure 7:**
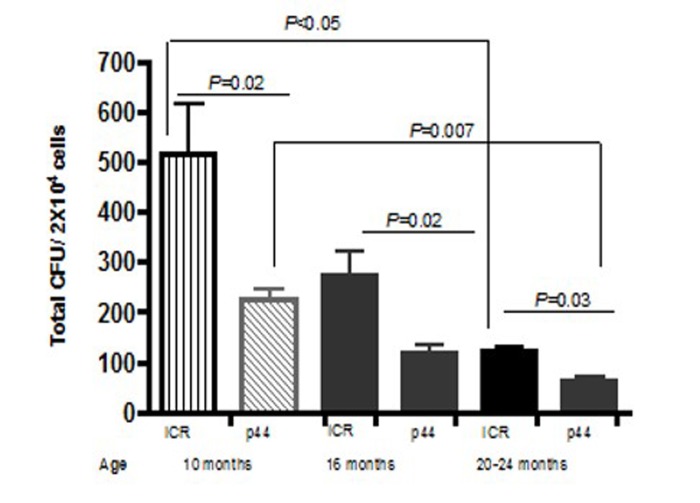
Total CFU decrease as mice age BM was isolated from at least 3 male ICR or p44 mice a 10, 16, or 20–24 months and 2 × 10^4^ cells were plated on MethoCult (StemCell Technologies). Colonies, defined as a cluster of at least 30 cells, were counted on Day 7. Results are mean ± SE (n≥3).

Based upon the above data demonstrating that recipients of old wild-type BM exhibit a significant reduction in survival as compared to recipients of young BM, we set up the following five experimental groups: six- to eight-week old female ICR recipient mice were lethally irradiated and transplanted with 1–2 × 10^6^ BMCs isolated from the following donors: 1) young (4-week-old) male p44 transgenic mice; 2) young male ICR mice; 3) old (11-month-old) male p44 transgenic mice; 4) 11 month old male ICR mice; and 5) un-transplanted females as controls. The numbers of animals used in each group were based upon the same statistical rationale as was used in the p53+/m experiments. As shown in Figure 6, BM from 4 week old wild type donors gave better short term survival (92%; 23/25) than recipients of 11 month old wild type BM 78%; 18/23). BM from p44 donors at both ages was less able to support short term survival than BM from wild-type donors: 4-week old donors (80%; 20/25) and BM from 11-month old p44 donors exhibiting poorest four-week survival (61%; 14/25). To determine whether these differences were due to p44 or age associated defects, we examined the number of total colony forming units (CFU) in BM isolated from ICR and p44 mice at 10, 16, and 20–24 months of age. As shown in Figure 7, BM from p44 mice had fewer CFU than BM from age-matched ICR controls. In addition, the number of CFU in BM of wild-type mice decreased significantly with aging (compare 10 months to 20–24 months; p<0.05). Taken together, these results indicate that both aging and p44 reduce the function and/or number of ST-HSCs.

### p44 premature aging model: long-term studies

**Figure 8:**
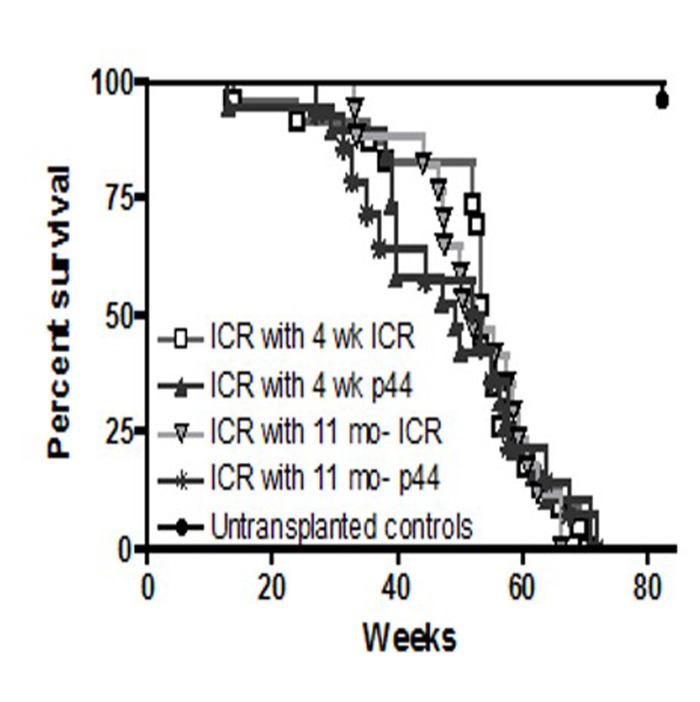
Long-term survival of ICR recipients

**Figure 9:**
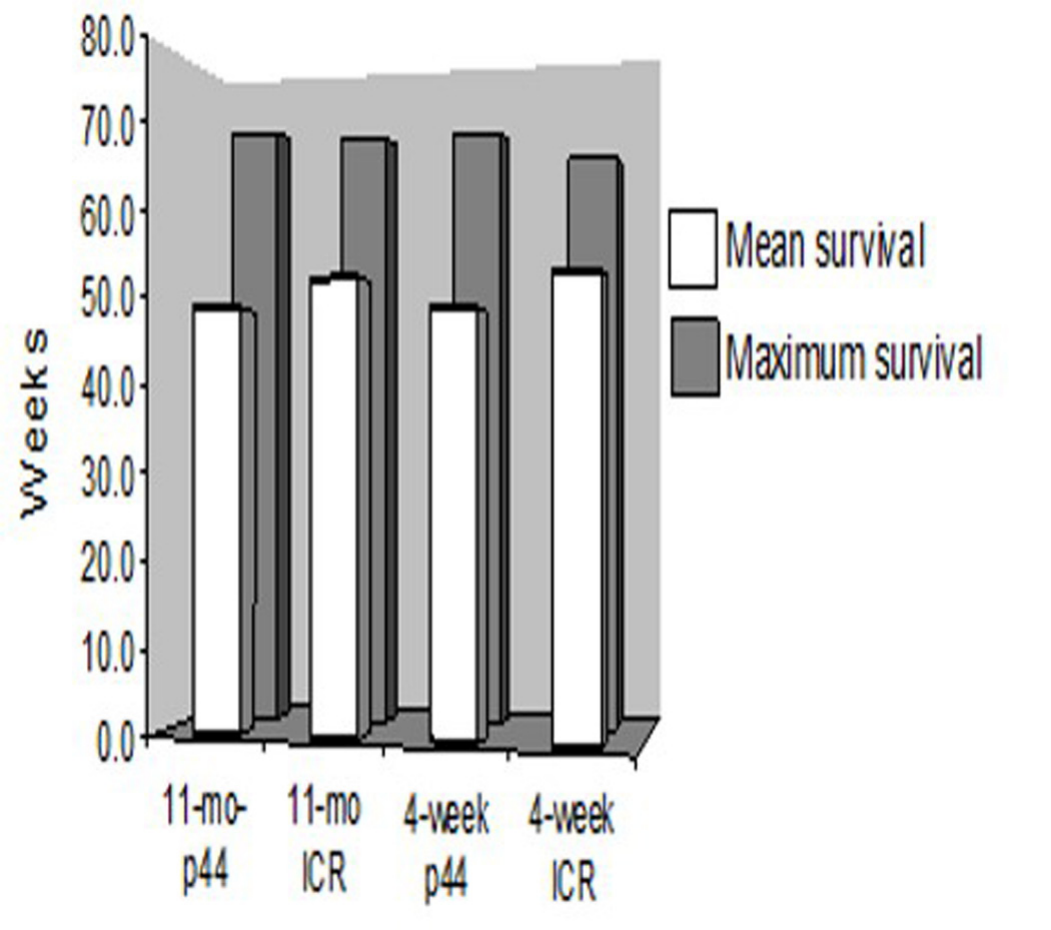
Mean and maximal life spans of ICR recipients Long term survival was similar across all transplanted groups of mice. Mean and maximum survival rates across all transplanted groups were approximately 50 and 70 weeks, respectively.

**Figure 10:**
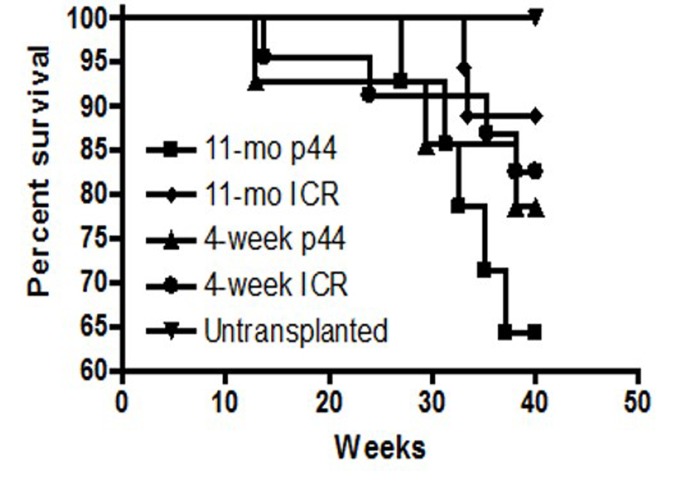
Survival analysis of BM transplant recipients at 40 weeks Recipients that received BM from 4-week old or 11-month old p44 donors exhibited a significant (p<0.05) reduction in mid-life survival as compared to un-transplanted controls or recipients of old or young wild-type BM.

Long-term survival was monitored in those mice that engrafted i.e. survived post-irradiation and transplantation for more than 4 weeks. Examination of the Kaplan-Meier survival curve demonstrated that the death rates among groups are almost identical after 50 weeks (Figure 8). Mean and maximum survival rates across all transplanted groups were 50 and 70 weeks, respectively (Figure 9). However, a post-exploratory analysis of survival through 40 weeks (Figure 10) demonstrates that recipients of BM from p44 donors (4-week and 11-month) had a significantly reduced mid-life survival as compared to the un-transplanted controls. In addition to defects in engraftment and CFU, transplantation of BM from old p44 mice also results in deficiencies that are manifested between 30 and 40 weeks of age (mid-life).

## DISCUSSION

### Stem cells and aging

Many hypotheses have been put forward to explain the aging process. One is that aging results from a defect in maintaining tissue homeostasis after stress [16]. According to this hypothesis, it is assumed that cells are constantly being lost due to continued intrinsic damage, such as that caused by generation of reactive oxygen species (ROS) that accompanies normal mitochondrial metabolism [17]. Additional alterations, including changes in DNA methylation of specific genes; especially those with CpG islands. These correlate with aging and may lead to transcriptional suppression of various genes [18]. Another hypothesis is that of antagonistic pleiotropy, wherein genes that enhance early survival and function, are disadvantageous later in life and may contribute to premature aging if not selectively suppressed in tissue renewing stem cells [19]. Regardless of the molecular mechanisms, terminally differentiated or damaged cells must be constantly replaced by proliferation of progenitor cells in the tissues. Aging may result when these cells, the tissue determined progenitor cells, are unable to keep up with the number or type of cells required for tissue renewal. Aging HSCs demonstrate a decrease in functionality, which is offset by an increase in overall number. However, in transplantation experiments using aged HSCs, these cells give rise to increased numbers of myeloid cells at the expense of lymphopoiesis [20–22]. This bias towards myeloid cells underlies the propensity of myelogenous diseases in the elderly [23]. Furthermore, studies of gene expression in aged HSCs have shown an increase in those genes whose products would contribute to increased inflammation, stress response and genomic instability [24–27] . For example, chromosomal CORE (center of regulated expression) analysis suggests that decreased transcriptional fidelity accompanies aging [28]. While HSCs are certainly necessary for replenishment of the blood and immune systems, they may also serve as precursors for tissue determined stem cells of other organs, in which case it is possible that they may contribute to the organismal aging [29–31] . We observed that recipients of both old and young wild-type BM displayed significantly reduced long-term survival compared to unirradiated controls. In addition, we could not identify bone marrow derived contribution to any other cell lineage than hematopoietic. There is some published evidence that bone marrow derived cells can contribute to other lineages, such as liver, but this is a very rare event [32,33].

### Aging and the stem cell niche

The role that the stem cell “niche” or “the microenvironment,” plays in stem cell aging is a subject of great current interest. For example, in the thymus, it has been shown that the thymic involution that accompanies aging in mice is due to extrinsic factors (reduced secretion of IL-7 by thymic epithelial cells [34]). Although recent results using transplantation models support a role for intrinsic changes in older HSCs [21, 35], other studies show that an age-altered microenvironment results in functional changes in HSCs and modulation of hematopoietic differentiation. HSCs from young mice have a reduced differentiation potential towards lymphocyte phenotype in aged compared to young recipients, and similarly, HSCs from aged mice also exhibit a reduced lymphoid differentiation potential in young mice [36]. HSCs from older mice demonstrate decreased adhesion within the bone marrow niche and increased mobilization in response to granulocyte-colony-stimulating factor (G-CSF) compared to young mice [37, 38]. Furthermore, homing of transplanted HSCs is reduced in old as compared to young recipients [22, 37, 38] . More recently, Ju et al. [39] demonstrated that transplantation of normal HSCs into telomerase knockout mice limited engraftment, further supporting a contribution of the niche to stem cell aging.

### HSC lineage development

Virtually all bone marrow derived HSC activity has been shown to be contained within the lineage–/lo(Lin–/lo) Sca1+ckit1+ (LSK) HSC compartment [40], which represents approximately 0.05% to 0.1% of total murine BM cells [41]. The prevailing model of hematopoiesis suggests that the LSK HSC pool consists of at least 2 functionally distinct HSC subpopulations, long-term and short-term repopulating HSCs (LT-HSCs and ST-HSCs, respectively). LT-HSCs have extensive (life-long) self-renewing potential and on commitment give rise to ST-HSCs with more restricted self-renewing capacity [42]. Thus, LT-HSCs are both required and sufficient to secure life-long reconstitution of the entire hematopoietic system following transplantation procedures in lethally irradiated recipients. However, in the first four weeks after recipients are irradiated, rapid and efficient replacement of short-lived myeloerythroid cell lineages is critical to overcome life-threatening cytopenia, and is thought to be dependent upon ST-HSCs. Our data suggest that recipients of p44 BM cells suffer from decreased short-term and mid-life survival, implicating deficiencies in the ST-HSC pool.

### p53 and aging

Cancer has been described as a disease of the elderly based on the observation that most tumors appear in the last quarter of life [43]. This is attributed to acquisition of mutations in genes encoding for oncogenes or tumor suppressors, 50% of which are associated with mutation of the p53 gene. Studies by Feng et al. suggest that diminished DNA repair, dysregulation of cell cycle regulation and subsequent tumorigenesis exhibited by older organisms is partly attributable to a decrease in p53 function in aged animals [44]. Thus, tissues from old mice exposed to gamma irradiation have a decline in p53 activity and stability compared to those from young irradiated mice. Furthermore, apoptosis is significantly reduced in the spleens of the irradiated old mice when compared to young mice.

### p53 models of premature aging: p53 +/m mice

Early studies of the role of p53 in aging were hampered as mice lacking p53 (p53−/−) develop lethal tumors early in life [45]. However, Tyner et al. demonstrated that mutant mice expressing a truncated form of *p53* (designated as p53m or the m-allele), display a premature aging phenotype [6]. This was the first evidence that p53 was involved in the aging process. The p53m isoform expresses five C-terminal exons of p53 and lacks the N-terminal sixth exon, as well as a large, undefined region upstream of the *p53* gene. Mice co-expressing the p53m isoform with wild-type (termed p53+/m) show enhanced resistance to tumorigenesis, reduced longevity, osteoporosis, hyper-responsiveness to stress induced by ionizing radiation and anesthesia, and reduced organ mass of muscle, spleen, liver, kidney and testes [6]. These mice display a diminished ability to regenerate peripheral white blood cells after ablation of hematopoietic progenitors with 5-fluorouracil, suggesting a defect in the ability of HSC functionality. The p53+/m mice also exhibit a five to 10-fold increase in overall HSCs number, but a three-fold loss in functionality and a decrease in engraftment capability when compared to aged matched 18 to 20 month-old control mice [46, 47]. In our original proposal we planned to use this model to determine if the aging phenotype might be due to changes in HSCs. However, as discussed above, this model did not work out for several reasons, and we changed to another p53 related model of aging, p44.

### p53 models of premature aging: p44 mice

The involvement of p53 in aging is further substantiated by development of the p53 transgenic mouse line (designated as p44), which contains a well-defined 3-kb deletion in exon 2, as well as 5′ and 3′ flanking intron sequences of p53, generating a 44 kDa protein [9]. This short form of p53, which is naturally occurring and was first reported in isolates of spleen cells from Friend virus–infected mice, utilizes an alternate translational initiation site in exon 4 and likely functions as a transient, negative regulator during cell cycle progression [48–50]. p44 mice exhibit shortened life spans, infertility, osteoporosis and slower growth rates. Mouse embryonic fibroblasts (MEFs) isolated from p44 mice fail to undergo the proliferative burst at passage one (P1), a characteristic of early passage wild-type MEFs. In contrast to p53+/m and p44 mutants, mice that overexpress p53 due to reduced expression of Mdm2 or increased expression of wild-type p53 show enhanced protection from tumor formation, without a decrease in normal lifespan [51, 52]. Expression of the p44 isoform leads to an enhancement of some p53 functions, as indicated by increased expression of p53 targets such as p21, MDM2 and insulin-like growth factor binding protein 3 (IGFBP-3). However, p44 effects on other p53 targets such as GADD45 demonstrate that expression of the p44 isoform results in diminished p53 function. It is likely that the dual properties, i.e., both increases and decreases in p53 functions, lead to the premature aging phenotype in p44 mice. In conclusion, although it is difficult to state the mechanisms, our BM transplantation results clearly show that recipients of BM from young mice survive longer than recipients of BM from older mice and that BM from p44 aging mice when transplanted to wild-type mice led to a selective decrease in mid-life survival.

## METHODS

### Animals and animal care

Inbred mice (all on the C57Bl/6J background) and outbred ICR mice were purchased from Jackson Laboratory, Bar Harbor, Maine and Taconic Farms, Hudson, New York, respectively. p53+/m mice were obtained from Dr Larry Donehower and the p44 mice were obtained from Dr Heidi Scrabble’s laboratory at the Mayo Clinic and bred in our animal facility. All mice were acclimatized and housed in the Wadsworth Center’s animal facility under controlled temperature and humidity in a 12 hour light/dark cycle. Purina lab chow and water were available ad lib. Unless otherwise noted, all mice were 4–8 weeks old at the start of each study. All experimental protocols were approved by the Wadsworth Center Animal Care Committee according to Animal Welfare Assurance Number A3183–01 of the National Institutes of Health.

### Irradiation

Female mice were individually irradiated in a ^137^cesium source (Gammator Model M-38, Isomedix Inc., Parsippany, NJ) at a dose rate of 255 rads/minute (1050 rads total dose). Transplantation of bone marrow was performed within 2 hours of irradiation. Mice were immediately placed on Neomycin-treated water (Sigma; Catalogue # N6386; 1 gram/liter) for 4 weeks, at which time they were considered engrafted. Thus, group size at 4 weeks post irradiation may be smaller than initial group size due to loss of mice that failed to engraft.

### Bone marrow transplantation

Bone marrow cells were collected from male donor mice following sacrifice by CO_2_ asphyxiation/cervical dislocation. Femur and tibia bone cavities were flushed into sterile test tubes with RPMI/10% FBS using a 25 G needle and a 1 ml syringe. Cell clumps were gently broken up by pipetting and red blood cells were depleted by lysis in 0.83% ammonium chloride on ice for 5 minutes. Cell viability was checked by trypan blue exclusion. The average yield from 1 donor was 30 × 10^6^ cells. For injection into recipients, bone marrow cells (2 × 10^6^, except where noted) were suspended in Hank’s balanced salt solution (HBSS) and delivered intravenously in 100 microliters.

### Pathology

Following engraftment, weekly body weight measurements were collected and percentage survival recorded. All mice were subjected to a full autopsy. Organ and tissue sections were presented on glass slides for pathological examination and scoring by a board-certified pathologist (S.Sell).

### Colony forming units (CFU) Assay for stem cell number

HSC numbers in BM samples are assayed by collection of BM cells (BMCs) and plating of mononuclear cells in a methylcellulose based medium Methocult™ (Stem Cell Technologies Inc., Vancouver, BC, Canada). This contains 1% methylcellulose, 15% fetal bovine serum, 1% serum albumin, 10 μg/ml insulin, 200 μg/ml transferrin, 10^−4^ M 2-mercaptoethanol, 2 mM L-glutamine, 50 ng/ml stem cell factor, 10 ng/ml IL-3, and 3 units of erythropoietin. Each assay was run in duplicate, plating 2 × 10^4^ bone marrow mononuclear cells/ml in Methocult in 35 mm petri dishes. The dishes were incubated at 37°C, 5% CO_2_ in a humidified incubator Colonies of greater than 50 cells were scored and counted under bright field illumination for their hematopoietic identity such as CFU-erythrocyte, CFU-granulocyte and CFU-monocyte, based on characteristic morphology.

### Tissue processing

For tissue examination, tissue blocks from various organs were collected and fixed in freshly prepared 4% PFA. Following automatic processing and embedding in paraffin, 5 μm sections were placed on conventional glass slides for hematoxylin and eosin staining or for immuno-histochemical staining (see below).

### Immunohistochemistry

Detection of green fluorescent protein (GFP)-labeled cells was performed on 5 μm sections on glass slides. Slides were incubated overnight with primary antibody (goat anti-GFP, Rockland Immunochemicals, Limerick PA; catalogue # 600–101-215) at 1:1,000. Biotinylated donkey anti-goat antibody (Jackson Immunoresearch, West Grove, PA; catalogue # 705–065-003) and Extravidin-peroxidase (Sigma, St. Louis, MO., catalogue # E2886) were applied the following day (1:1,000 and 1:800, respectively). Color was developed with diaminobenzidine (Sigma; catalogue # D 8001) and slides counterstained with haematoxylin.

### In situ hybridization

Formalin-fixed, paraffin-embedded sections were deparaffinized through xylene washes and rehydrated through a graded ethanol series. A FITC-labeled probe for the mouse Ychromosome (catalogue # 1189-YMF-01; Cambio Ltd., Cambridge, UK) was hybridized to pepsin-treated sections for 10 minutes at 60°C, followed by overnight incubation at 37°C, according to the manufacturer’s directions. Amplification of the probe was performed by primary (goat anti-fluorescein; Rockland, Gilbertsville, PA., catalogue # 600–101-096) and secondary (fluorescein-conjugated donkey anti-goat; Jackson Immunoresearch, West Grove, PA., catalogue number 705–095-147) antibody enhancement. Nuclei were stained with 4′,6-diamidine-2-phenylindole dihydrochloride (catalogue # 236276; Roche Applied Sciences, Indianapolis, IN.). Images were captured on an Olympus BX 51 microscope equipped with fluorescence detection and Optronics PictureFrame Version 1.2 software.

### Statistical analysis

A comprehensive statistical analysis was performed by Dr. Andrew Reilly of the Wadsworth Biostatistics Core Facility as well as by Tairan Ye and Dr. James Grady of U. of Connecticut Health Center. The rationale for the numbers of mice in each group to provide the power to obtain significant results is based on an analysis of the survival data described in Tyner et al. [6]. Assuming that the survival curves of the major experimental groups will be on the same order as described, a minimum number of 18 mice in each group give a power of > 99%.

## SUPPLEMENTAL DATA FIGURES


