# Supplementary material for: Survival of irradiated recipient mice after transplantation of bone marrow from young, old and “early aging” mice
Source: Aging (Albany NY). 2015 Dec 30;7(12):1212–22. doi: 10.18632/aging.100867 (PMC4712343; doi:10.18632/aging.100867)
Supplement: Supplementary file 1 [file aging-07-1212-s001.pdf]

SUPPLEMENTAL DATA

Please browse the link in Full Text version to find the Supplemental Data related to this manuscript.

SUPPLEMENTAL FIGURES

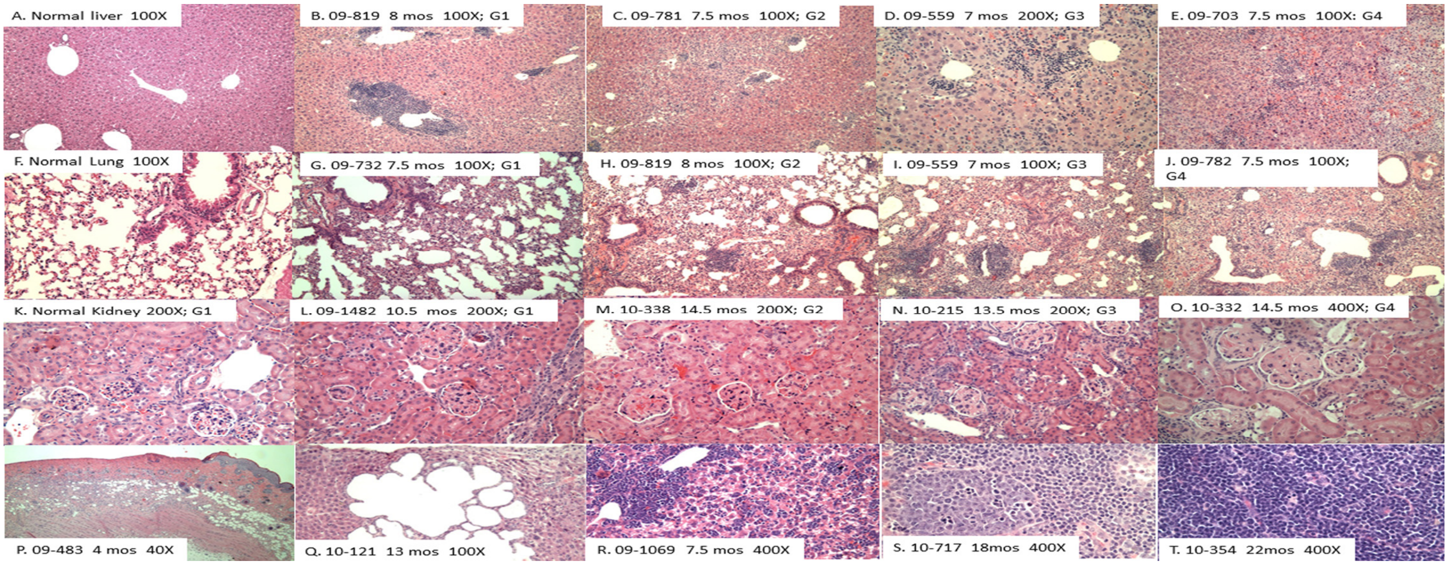

Supplement 2. Grading of hepatitis (**A-E**), Pulmonary inflammation (**F-I**), Glomerulosclerosis (**K-O**) and miscellaneous lesions in aging mice (**P-T**). Each photomicrograph has the microscopic slide number and magnification. The grade (magnitude) of the lesions varies from 1 (**G1**) to 4 (**G4**) is also indicated for hepatitis (**A-E**, lung lesions (**F to J**) and glomerulosclerosis (**K-Q**). The liver lesions vary from focal collections of lymphocytes (**B**) to increasing involvement of the liver parenchyma with infiltrating mononuclear cells and hepatocyte necrosis (**C-E**). The lung show increasing large mononuclear cell infiltration of the alveoli, focal lymphocytic nodules and hyperplasia of bronchial mucosa **G-J**). There is increasing sclerosis of the renal glomeruli with hyperplasia of Bowman's capsule **L-O**). Other lesions are hyperplasia and ulcerating chronic inflammation of the skin (Barbering, **P**), many mice including non-irradiated controls; peliosis hepatis, one mouse (**Q**); leukemia (2 mice; recipients of 7 week old bone marrow) (**R**); adenocarcinoma of salivary gland (control mouse, 18 months old, S); and non-nodular lymphoma (control mouse, 14.5 months old, (**T**).

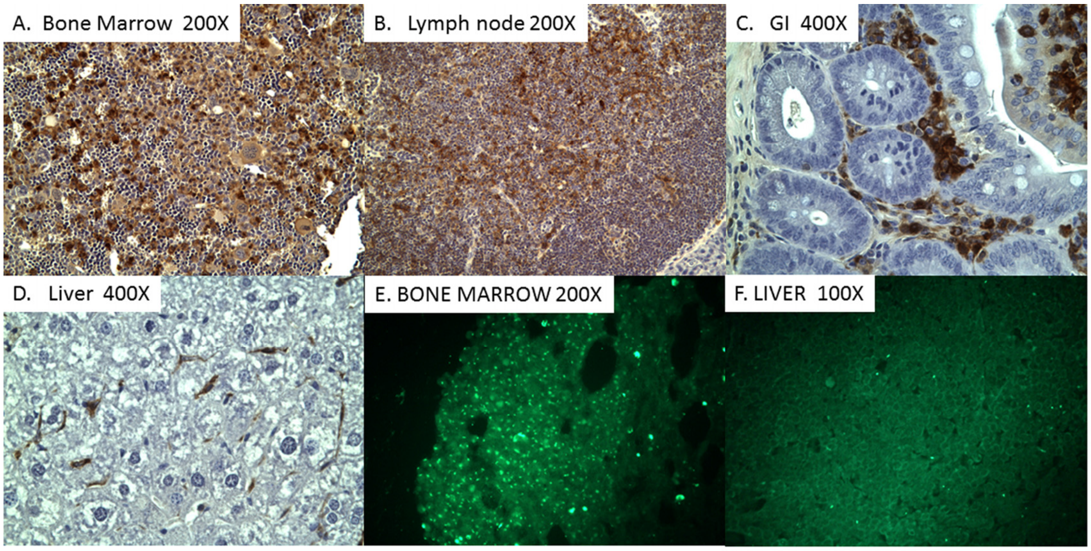

Supplement 3. Immunoperoxidase labeling for GFP; **E, F**. Fluorescence labeling for Y-chromosome. There are many GFP and Y-chromosome positive cells in the bone marrow (**A,E**), medulla of the spleen (**B**) and the core mesenchymal cells of the intestinal villi (**C**). The sinusoidal cells of the liver (**D,F**), but not epithelial cells, are labeled.
